# Downregulation of miR-92a Is Associated with Aggressive Breast Cancer Features and Increased Tumour Macrophage Infiltration

**DOI:** 10.1371/journal.pone.0036051

**Published:** 2012-04-26

**Authors:** Sofie Nilsson, Christina Möller, Karin Jirström, Alexander Lee, Susann Busch, Rebecca Lamb, Göran Landberg

**Affiliations:** 1 Center for Molecular Pathology, Department of Laboratory Medicine, Lund University, Skåne University Hospital, Malmö, Sweden; 2 Pathology, Department of Clinical Sciences, Lund University, Skåne University Hospital, Lund, Sweden; 3 Breakthrough Breast Cancer Research Unit, School of Cancer and Enabling Sciences, University of Manchester, Manchester Academic Health Science Centre, Paterson Institute for Cancer Research, The Christie NHS Foundation Trust, Manchester, United Kingdom; Karolinska Institute, Sweden

## Abstract

**Background:**

MicroRNAs are small non-coding RNAs involved in the regulation of gene expression on a posttranscriptional level. These regulatory RNAs have been implicated in numerous cellular processes and are further deregulated in different cancer types, including breast cancer. MiR-92a is part of the miR-17∼92 cluster, which was first reported to be linked to tumourigenesis. However, little is known about the expression of miR-92a in breast cancer and potential associations to tumour properties. The expression of miR-92a was therefore characterized in 144 invasive breast cancer samples using *in situ* hybridization and related to clinico-pathological data as well as to selected key properties of the tumour stroma, including the presence of macrophages (CD68) and cancer activated fibroblasts (alpha-SMA).

**Methodology/Principal Findings:**

To measure miR-92a levels, an *in situ* hybridisation protocol was developed and validated using cell lines and miR-92a inhibitors. The expression in the tumour samples was objectively evaluated using digital image analysis program subtracting background activities. We found that the miR-92a expression varied between tumours and was inversely correlated to tumour grade (r = −0.276, *p* = 0.003) and recurrence-free survival (*p* = 0.008) and provided independent prognostic information in multivariate Cox analysis (HR: 0.375, CI: 0.145–0.972, *p* = 0.043). MiR-92a was moreover inversely correlated to the number of infiltrating macrophages in the tumour stroma (r = −0.357, *p*<0.001), and downregulation of miR-92a promoted cell migration (*p*<0.01).

**Conclusions/Significance:**

This study demonstrates that downregulation of miR-92a in breast cancer is linked to key epithelial and stromal properties as well as clinical outcome.

## Introduction

MiRNAs (microRNAs) are a class of non-coding endogenously expressed RNAs that regulate mRNAs on a posttranscriptional level. These short (∼22 nucleotides) gene regulatory RNA molecules are involved in numerous cellular processes including proliferation, metabolism, cell death, cell motility and development [1]. MiRNAs have also been linked to cancer, mainly due to proliferation and apoptosis regulatory functions for some miRNAs, but also based on the localization of miRNAs to fragile sites in the genome [2]. MiRNA expression profiles that can further distinguish between normal breast tissue and breast cancer have been presented by several groups [3], [4]. These differences in expression for certain miRNAs in breast cancer suggests that miRNAs can be useful biomarkers for breast cancer progression and clinical course or might even be key molecular events in the transformation process. Since each miRNA is believed to regulate multiple mRNAs, alterations of one miRNA will have impact on several target genes in a number of different pathways, suggesting that it is more efficient to target or monitor a single miRNA than one individual mRNA [5].

MiR-92a is part of the miR-17∼92 cluster, consisting of seven miRNAs (miR-17-5p, miR-17-3p, miR-18a, miR-19a, miR-19b, miR-20a and miR-92a), first described as an oncogenic miRNA cluster involved in B-cell lymphoma [6]. However, other studies have reported contrasting roles for these miRNAs, both as a cluster and as individually miRNAs [7]. Among the members in the cluster, miR-92a is the least characterized subunit. A potential link to tumourigenesis has originated from studies of miR-92a levels in the plasma of patients with different types of malignancies. In acute leukemia, plasma level of miR-92a could successfully distinguish between leukemic patients and healthy donors [8], and similar observations have been described in colorectal cancer [9]. In patients with hepatocellular carcinoma, miR-92a has been reported to be overexpressed in the cancerous tissue whereas levels of miRNA in plasma were lower compared to healthy donors [10]. The role of miR-92a in breast cancer has not been fully clarified and a relationship to the miR-17∼92a cluster does not necessarily suggest that all these miRs have oncogenic properties. Zhang *et al* also demonstrated that the miR-17∼92a cluster was deleted in 21.9% of analyzed breast cancer samples, indicating that miRNA alterations can differ depending on cancer type [11].

In this study we developed an *in situ* hybridization method for the detection of miR-92a in formalin fixed tumour material and investigated the expression in a tissue microarray consisting of 144 breast cancer samples. Objective digital image analysis was used to delineate mir-92a expression and interestingly downregulation of miR-92a was inversely associated to tumour grade, recurrence-free survival and the presence of tumour infiltrating macrophages. *In vitro* studies also revealed an increase in cell migration after downregulation of miR-92a. These novel data suggest a possible interaction between epithelial miR-92a levels and key tumour properties and aggressiveness as well as links to immune cells in the tumour stroma.

## Results

### Validation of the miR-92a *in situ* hybridization method

**Figure 1:**
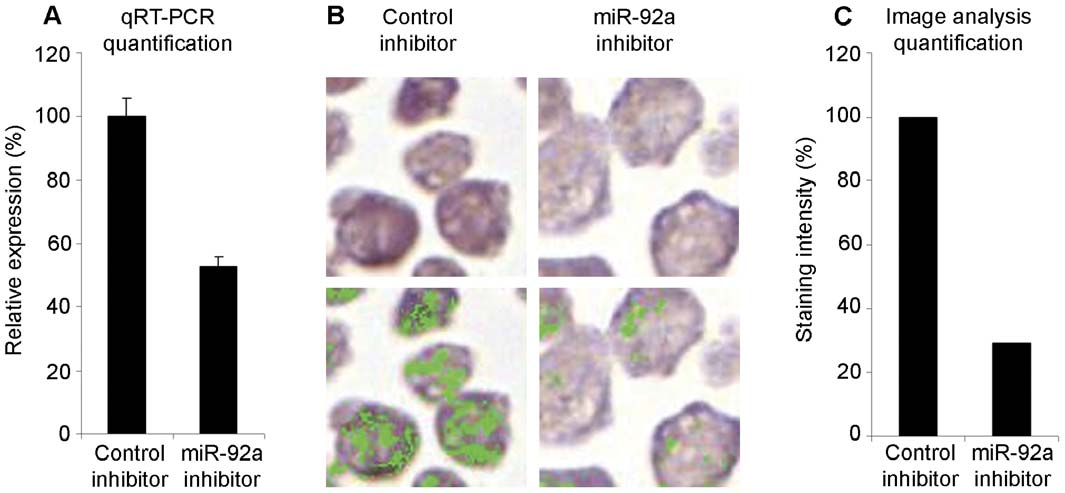
Validation of miR-92a *in situ* hybridization probe. *A)* Downregulated of miR-92a in MDA-231 cells was validated and confirmed by qRT-PCR. *B)* Detection of miR-92a using *in situ* hybridization in MDA-231 cells after downregulation of miR-92a. Lower panel shows the definition of positive pixels identified by the digital image analysis program. *C)* Quantification of positive pixels by the image analysis program.

In order to validate the accuracy of the miR-92a detection probe, the breast cancer cell line MDA-231 was transfected with a miR-92a inhibitor and downregulation was confirmed by qRT-PCR (Figure 1A). *In situ* hybridization for miR-92a was then performed using the same batch of cells as for the qRT-PCR. Digital image analysis software was used to analyze the expression of miR-92a and confirmed a decreased expression after inhibitor treatment both visually and by quantification of the staining intensity using digital image analysis (Figure 1B–C).

### Expression of miR-92a in human breast and in breast cancer

**Figure 2:**
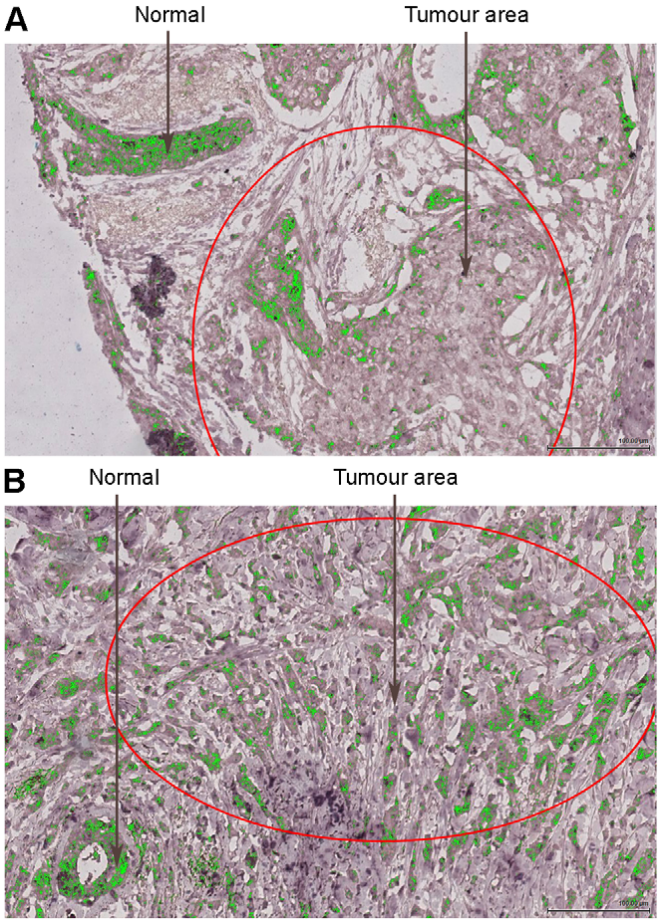
MiR-92a expression in normal breast and breast cancer samples. *A)* and *B)* Two examples of miR-92a expression in normal breast tissue compared to tumour areas.

**Figure 3:**
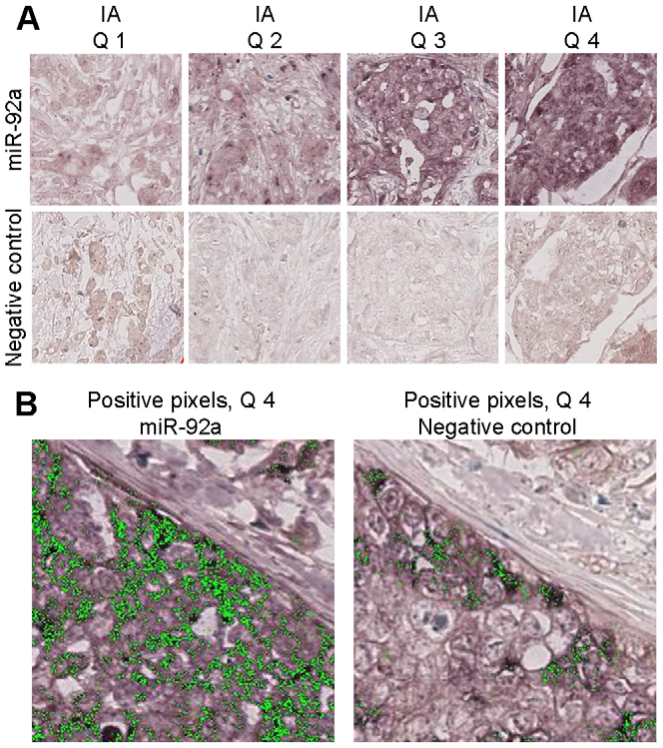
MiR-92a expression in breast cancer samples. *A) In situ* hybridization was performed on a breast cancer tissue microarray using a detection probe for miR-92a and a negative control probe. Tumours were divided into quartiles 1–4 (Q1–Q4) based on the staining intensity (Q1 = weakest staining, Q4 = strongest staining). 20× magnification. *B)* Example of positive pixel definition of representative Q4 tumour stained with miR-92a probe and negative control probe. 40× magnification.

We next performed miR-92a *in situ* hybridization of a TMA (tissue microarray) containing 144 breast cancer samples [12]. Figure 2 illustrates the expression of miR-92a in normal breast and cancer tissue. The staining shows a higher expression of the miRNA in the normal tissue compared to tumour areas. To compensate for variations in background staining we used a negative control probe and hybridized it to a second set of the TMA. The signal from the negative control was then subtracted from the miR-92a expression signal for each tumour core, resulting in a representative miR-92a expression value. The digital image analysis was performed with the Digital Image Hub software from Slidepath and generated information about staining intensity and fraction positive cells in 117 (81%) breast cancer samples. The missing cases (n = 27), which did not contain enough tissue for analysis, did not differ significantly from the successful cases regarding age, tumour size and grade, lymph node status, Ki67, ER/PR/HER2 status and stroma markers (data not shown). The staining intensity and fraction positive cells for miR-92a were strongly correlated (Pearson's chi square correlation = 0.968, *p*<0.001) and only staining intensity values were used in the following analyses. Patients were further divided into quartiles according to expression of miR-92a where quartile 1 (Q1) had the lowest intensity and quartile 4 (Q4) the highest (Figure 3A). Hybridization with the miR-92a probe and the negative control probe of a Q4 tumour followed by image analyses detection is illustrated in Figure 3B.

### miR-92a and clinico-pathological properties and its cellular role

**Figure 4:**
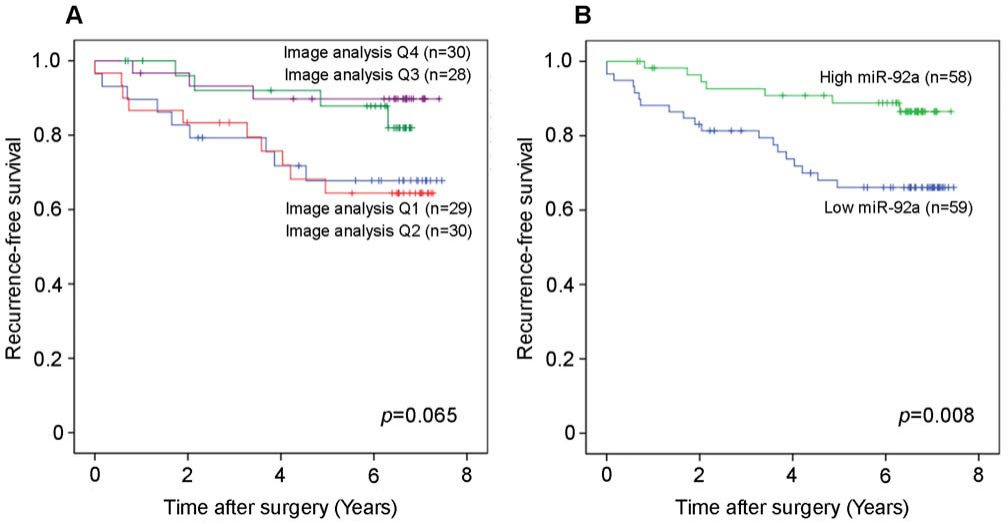
Recurrence-free survival among breast cancer patients according to miR-92a expression. *A)* Tumours were divided into four groups based on the staining intensity of miR-92a and recurrence-free survival for the patients was determined, *p* = 0.065. *B)* Recurrence-free survival analysis after merging of tumours in Q1–Q2 and Q3–Q4, *p* = 0.008.

First, we investigated the association between miR-92a expression and established clinico-pathological parameters with a focus on epithelial tumour properties. As presented in table 1, miR-92a was significantly inversely correlated to tumour grade (Spearman's ρ = −0.276, *p* = 0.003) whereas there was no significant link to proliferation, ER-status, tumour size or lymph node positivity. We next analyzed the potential link between miR-92a and breast cancer recurrence. Kaplan Meier analysis clearly illustrates that the patients with the highest expression of miR-92a (Q3 and Q4) had a better outcome compared to patients with lower miR-92a expression (Q1 and Q2) expression (Figure 4A, *p* = 0.065). This difference in recurrence was highly significant when merging the two lowest quartiles into low miR-92a expression and the two highest into high miR-92a expression (*p* = 0.008) as presented in Figure 4B.

**Table 1:** Distribution of miR-92a expression in breast cancer tumours according to clinico-pathological parameters.

	miR-92a staining intensity				
	Q 1	Q 2	Q 3	Q 4	
Variable	N = 29	N = 30	N = 28	N = 30	*P* †
Median age, y (range)	59 (35–90)	67,5 (49–97)	66,5 (44–91)	62 (34–87)	0.337‡
Tumor size, mm					
≤20	13	11	16	19	0.065
≥21	16	19	12	11	
Lymph node status					
Negative	16	11	14	16	0.984
Positive	9	17	11	12	
NHG					
I	1	2	4	9	0.003
II	10	14	16	10	
III	18	14	8	11	
Ki67-positive, %					
0–10	2	2	0	1	0.523
11–25	6	11	14	10	
>25	17	14	10	15	
ER-positive, %					
<10	6	4	2	5	0.549
>10	23	26	26	25	
PR-positive, %					
<10	12	11	6	8	0.127
>10	17	19	22	22	
HER2 gene amplification					
No	24	28	26	28	0.160
Yes	4	2	2	1	
CD68+ cells (macrophages)					
Q 1	3	3	9	14	<0.001
Q 2	5	6	8	6	
Q 3	7	14	4	6	
Q 4	10	3	6	3	
SMA intensity					
1	1	5	2	1	0.885
2	18	15	16	17	
3	7	5	3	6	

† Correlations were calculated using Spearman's ρ(two-sided) unless otherwise specified. *P* values were not adjusted for multiple testing.

‡ Kruskal-Wallis test (two-sided).

In order to further investigate the potential prognostic role of miR-92a, we performed a univariate Cox proportional hazard regression analysis and observed a significant benefit for patients with tumours displaying high expression of miR-92a (*p* = 0.012, hazard ratio = 0.328, CI: 0.138–0.781) (Table 2). We also performed a multivariate Cox proportional hazard regression analysis adjusting for known prognostic factors as age, tumour size, lymph node status, tumour grade and ER-status. Even in this model, patients with high levels of miR-92a had a significantly (*p* = 0.043) lower risk of recurrence, with a hazard ratio of 0.375 (CI: 0.145–0.972), compared to the patients with low expression of miR-92a (Table 3). These data demonstrate that miR-92a adds independent prognostic information in breast cancer and patients with high levels of miR-92a have better clinical outcome than patients with low levels.

**Table 2:** Univariate Cox proportional hazard regression model of the risk of recurrence according to clinico-pathological factors and miR-92a expression.

		Recurrence-free survival		
Variable		HR	95% CI	*P*
Age	continuous	1.038	1.008–1.069	0.012
Tumour size	≤20 mm	1.0		
	≥21 mm	3.141	1.391–7.096	0.006
Lymph node status	negative	1.0		
	positive	6.701	2.523–17.795	<0.001
Tumour grade (NHG)	I+II	1.0		
	III	3.363	1.561–7.244	0.002
ER	<10%	1.0		
	>10%	0.275	0.125–0.604	0.001
miR-92a	Low	1.0		
	High	0.328	0.138–0.781	0.012

NHG = Nottingham Histological Grade; ER = estrogen receptor; HR = hazard ratio; CI = confidence interval.

**Table 3:** Multivariate Cox proportional hazard regression model of the risk of recurrence according to miR-92a expression adjusted for clinico-pathological factors.

		Recurrence-free survival		
Variable		HR	95% CI	*P*
Age	continuous	1.038	1.002–1.076	0.041
Tumour size	≤20 mm	1.0		
	≥21 mm	1.923	0.657–5.633	0.233
Lymph node status	negative	1.0		
	positive	5.686	1.863–17.358	0.002
Tumour grade (NHG)	I+II	1.0		
	III	0.774	0.252–2.371	0.653
ER	<10%	1.0		
	>10%	0.202	0.063–0.645	0.007
miR-92a	Low	1.0		
	High	0.375	0.145–0.972	0.043

NHG = Nottingham Histological Grade; ER = estrogen receptor; HR = hazard ratio; CI = confidence interval.

**Figure 5:**
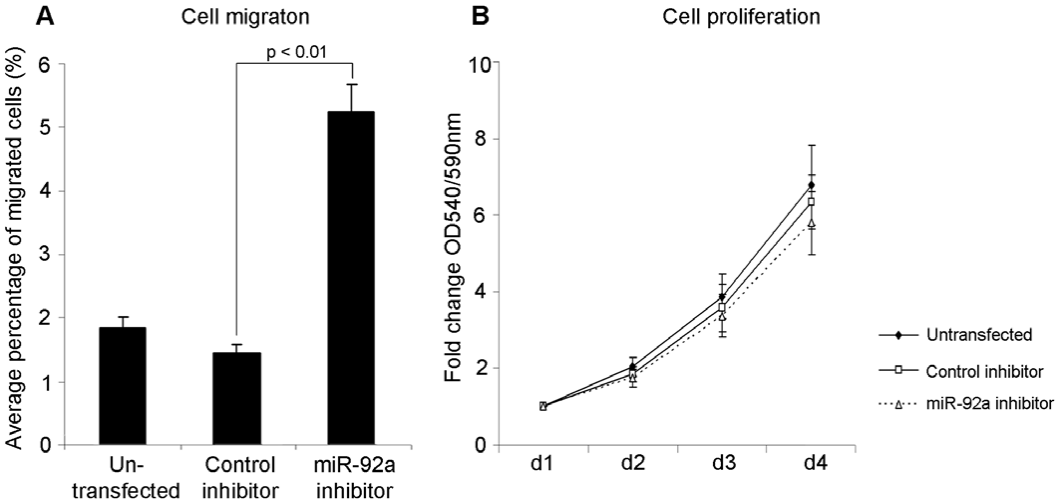
Cellular properties of miR-92a in breast cancer cell line MDA-231. Downregulation of miR-92a in MDA-231 cells led to in increased migration (*A*) but did not affect the proliferation of the cells (*B*).

Inhibition of mi92a in the breast cancer cell line MDA-MB-231 in comparison to control cells showed a highly significant increase in migration (p<0.01) which was not caused by increased proliferation (Figure 5). These data may provide a mechanism to support our observations within primary breast cancer samples whereby loss of miR-92a was associated with poor clinical outcome.

### miR-92a and tumour stroma properties

**Figure 6:**
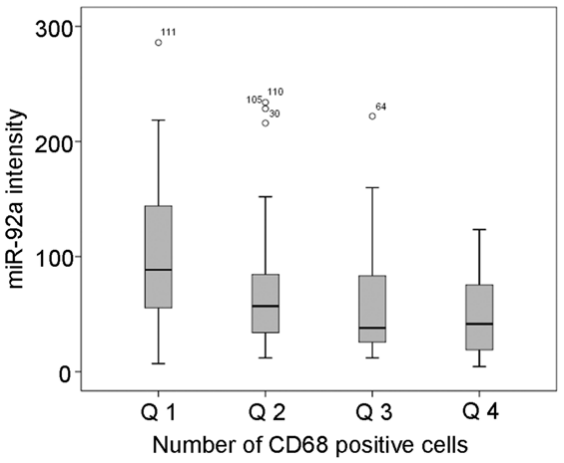
Inverse link between CD68-positive macrophages and miR-92a expression. Tumours were divided into quartiles based on number of CD68-positive cells (macrophages) and compared to miR-92a staining intensity.

We next investigated the association between miR-92a and a selection of key malignant properties within the tumour stromal compartment, e.g. the presence of cancer activated fibroblasts and macrophages. Activated fibroblasts were defined as smooth muscle actin α (SMAα) positive and macrophages were defined as CD68-positive cells. There was no link between miR-92a and SMA-positivity in fibroblasts (*p* = 0.962) but interestingly a strong inverse link to macrophage content was observed (Spearman's ρ = −0.357, *p*<0.001) (Table 1). As illustrated in figure 6, the lowest quartile of CD68-positive tumours had a substantially higher miR-92a content compared to a stepwise reduction in miR-92a levels with increasing CD68 macrophage contents indicating an inverse link between miR-92a expression and macrophages. This suggests a role for miR-92a in the interaction between epithelial cells and immune cells in the tumour stroma compartment.

## Discussion

In this report we have developed and validated an *in situ* hybridization method for the detection of miR-92a in breast cancer samples and successfully linked its expression levels in breast cancer to several key tumour properties. *In situ* hybridization is a powerful method for the detection and localization of a specific nucleotide sequence within a preserved cellular and tissue context, however the method is often debated due to issues with specificity and demanding optimization procedures [13]. Our results nevertheless clearly confirm that this approach, although challenging, can be valuable for the detection of miRNA in formalin fixed material of cancer samples arranged in TMAs.

One issue with the detection of miRNA using *in situ* hybridization is the length of the detection probe. For mRNA *in situ* hybridization, an oligonucleotide probe is generally 40–50 base pairs long. A miRNA is approximately 22 nucleotides long which makes it unfeasible to use a standard oligonucleotide probe [13]. Therefore, we alternatively used LNA (locked nucleic acid)-enhanced microRNA detection probes. These probes are exactly complimentary to the target miRNA and the incorporated LNA molecules leads to a stable probe:miRNA duplex increasing the success rate of the detection. Another concern is the amount of the target miRNA present in the tissue. Low amounts of specific miRNAs are difficult to detect due to loss of miRNAs by diffusion out of the tissue sections during the preparation of the tissue. In this study, the retention of miRNAs was improved by introducing an additional fixation step to the procedure using 1-ethyl-3-(3-dimethylaminopropyl) carbodiimide (EDC), which creates an irreversible cross-link between the 5′ phosphate of the miRNA and the amino groups in the protein side chains [14]. To further improve the detection of low abundant miRNAs, we used double-DIG labelled probes in order to enhance the detection signal. Even though we have developed a functional miRNA *in situ* hybridization protocol for miR-92a, we were unable to obtain successful results for two additional miRNA probes (data not shown), elucidating the challenges associated with the method.

For validation, *in situ* hybridization probes were tested in cells transfected with a specific inhibitor targeting the candidate miRNA and only probes displaying a clear reduction in the *in situ* signal in parallel with a miRNA decrease by qRT-PCR were selected for further analysis and were considered to be specific. In order to further assure the accuracy of the measured miRNA expression in the tissue, we normalised the detected miR-92a probe signal by subtracting a background signal. To do so, a second set of TMAs were hybridized with a control probe which is not complementary to any known microRNA and thus would not specifically bind to the tissue. Any detected signal therefore indicates background noise. The use of proper controls and evaluating the expression intensity objectively with digital image analysis secured the quality and reliability of the miRNA content analyses in the tissue specimens in our study.

The expression pattern of miRNA is complex and seems to be cell and tissue specific and also varies in different malignancies [15]. One specific miRNA can have diverse expression levels in different types of tumours probably depending on the tissue and origin of the tumour (tumour properties). Also the regulation of miRNA expression itself may be aberrant in some tumours. MiR-125b was shown to be lost in breast cancer [3] whereas it is frequently upregulated in pancreatic cancer [16]. Similar contrasting results for other malignancies and miRNAs seem likely and it is therefore important to carefully clarify the expression of various miRNAs in specific tumour types separately. MiR-92a is part of a reported oncogenic miRNA cluster and has been demonstrated to be upregulated in hepatocellular carcinoma [6], [10]. However, it has also been reported that the genomic region, in which miR-92a is located, is often deleted in breast cancer [11]. Here we observed that miR-92a expression varied amongst different breast cancer samples. Tumours displaying low miR-92a levels were associated with high tumour stage and poor recurrence-free survival suggesting that miR-92a is a potential marker for tumour progression. In fact, multivariate analysis including age, tumour size and stage, lymph node and ER status revealed that miR-92a is an independent prognostic marker.

Additionally we have observed that miR-92a is present in normal breast ducts and lobular units suggesting that miR-92a plays a physiological role in the normal breast tissue and is most likely downregulated in a fraction of breast cancer. The functional consequences of a loss of miR-92a have previously not been described. In this study we observed that downregulation of miR-92a in breast cancer cells resulted in increased cell migration, and it has been predicted that miR-92a affect genes that are involved in the regulation of the actin cytoskeleton and MAPK signalling pathway. Both processes are well known to be associated with cancer [17].

In addition to the link to tumour differentiation and disease recurrences, miR-92a was strongly and inversely correlated to the amount of infiltrating macrophages in the tumour stroma. It is becoming evident that the interplay between the components of the stromal compartment and the epithelial cells is of great importance and might influence both tumour initiation and progression [18]. Bandres *et al* demonstrated that miR-451 was directly targeting the pro-inflammatory cytokine macrophage migration inhibitory factor (MIF), which activates several macrophage functions [19], [20]. This potential crosstalk between miRNA expression in the epithelial cells and the response in cells located in the surrounding stroma is potentially relevant for miR-92a. Whether miR-92a has a gene target linked to macrophage infiltration remains to be further investigated.

In summary, in this study we present entirely novel data about the expression of miR-92a in breast cancer using an in situ hybridization approach. For the first time we show that miR-92a is linked to tumour stage and disease-free survival and although patient cohort was small, miR-92a expression provided independent prognostic information in a multivariate analysis. Strikingly, miR92a expression was also associated with macrophage infiltration indicating an important role of miRNAs in tumour-stromal interactions.

## Materials and Methods

### Ethics Statement

The study was approved by the Ethics Committee at Lund University (reference no 447-07) in Lund, Sweden. The ethics committee considered that informed consent was not to be required other than by the opt-out method. The data was analyzed anonymously.

### Patient material

The breast cancer specimens used in this study were obtained from 144 patients undergoing surgical resection at Malmö University Hospital, Malmö, Sweden, between 2001 and 2002. The median age at diagnosis was 65 years, and the median follow-up time for disease-specific and overall survival was 78 months. 31 (21%) of all patients in this cohort received adjuvant chemotherapy. Additional clinical information has been described previously [12], [21]. The study was approved by the regional ethical committee in Lund, Sweden.

### Cell culture and transfection

#### Validation of *in situ* hybridization probe

The breast cancer cell line MDA-MB-231 was maintained at 37°C in a humidified atmosphere with 5% CO_2_ and grown in RPMI 1640 growth medium supplemented with 10% fetal bovine serum (FBS), 1% sodium pyruvate and 1% penicillin and streptomycin. Cells were seeded 24 h before transfection. Lipofectamine 2000 (Invitrogen, CA, USA) was used for transfection of 20 nM miRNA Inhibitor (Exiqon, Vedbaek, Denmark) into the cells according to the manufacturer's instructions. The growth medium was changed to serum-containing medium 6 h after transfection. Cells were harvested 48 h after transfection for migration, proliferation and RNA extraction for qRT-PCR validation as well as cells were fixed and embedded in paraffin for validation of the *in situ* hybridization probe. Total RNA, including small RNAs, was extracted using the miRNeasy kit (Qiagen, Sollentuna, Sweden) according to the manufacturer's instructions with the exception of using 70 µl BCP (1-bromo-3-chloro-propan) instead of choloform. RNA concentrations and purity, measured by the ratio γ260/γ280 (2.08–2.09) were verified by measuring absorbance on the NanoDrop Spectrophotometer ND-1000. 500 ng of RNA was reverse-transcribed using Megaplex Primer Pool A. Individual TaqMan MicroRNA Assays (Applied Biosystems, CA, USA) were used for quantification of miR-92a and the endogenous controls RNU48 and MammU6 snRNA according to the manufacturer's instructions. The qRT-PCR was carried out using 7300 Real Time PCR Systems (Applied Biosystems) under the conditions: 10 min at 95°C followed by 40 cycles of 95°C for 15 sec and 60°C for 60 sec. The comparative threshold cycle (Ct) method was used for calculation of miRNA expression.

### Migration assays

Cell migration was routinely carried out in 8 µm-pore polycarbonate membrane Transwell chambers with a diameter of 6.5 mm (Corning, Inc. Corning, NY). The membranes were incubated in 150 µl serum-free RPMI 1640 for an initial equilibrium period of 1 h. Cells were resuspended in serum-free medium (1×10^6^ cells/ml) and 50,000 cells were added to each migration chamber. The chambers were placed into wells containing 600 µl 10% FCS medium and cells were allowed to migrate for 5 h following inhibition of mi92a. Cells remaining in the chamber were removed with a cotton swab and the migrated cells situated on the lower side of membranes were fixed for 15 min in PBS containing 4% paraformaldehyde. Membranes were cut and mounted on glass slides for DAPI staining and counted using a fluorescent microscope (cells were counted in 10×-magnification fields representing the composition of the membrane and the average percentage of cells determined). Assays were repeated three times.

### Proliferation assays

Growth curve was monitored using Alamar blue (Invitrogen). Cells were seeded out in 96 well plates at 2.5×10^4^/ml concentration (2500 cells/well) 48 h post-transfection. Cell viability was measured every 24 h for 4 days by adding 2% Alamar blue and fluorescence was read at 540/590 nm after 1 h incubation. Optical density values were normalised to day 1 reading.

### Tissue specimens and immunohistochemistry

All included cases were histopathologically re-evaluated and TMAs (tissue microarrays), containing duplicate 1.00 mm cores, were constructed from areas with invasive breast cancer using a tissue array machine (Beecher Instruments, Microarray Technology, MD, USA). Briefly, 4 µm thick TMA sections were mounted onto glass slides and deparaffinised followed by antigen retrieval using Dako's PTLink (DAKO, Glostrup, Denmark). Immunohistochemistry was then performed using Dako's Autostainerplus with the EnVisionFlex High pH-kit (DAKO) with the following antibodies: ERα (DAKO, M7047 1∶50), PR (DAKO M3569 1∶400), Ki-67 (DAKO M7240 1∶200), CD68 (DAKO M0876 1∶1500), αSMA (DAKO M0851 1∶1000). Detailed data on ER and PR analyses have been published previously [21]. CD68 was used as a pan-macrophage marker and the total amount of CD68-positive cells within the tumour area in the TMA cores were manually calculated. Alpha-SMA was used to detect cancer activated fibroblasts adjacent to invasive breast cancer cells and was scored according to the proportion of positive fibroblasts into three subgroups (negative to low, intermediate or high) with the following distribution; 9 (9%), 66 (69%), 21 (22%). All immunohistochemistry scoring was performed independently of a pathologist and a research associate without knowledge of pathological and clinical data.

### MiRNA *in situ* hybridization

FFPE (formalin-fixed paraffin-embedded) sections (4 µm) from the TMA were mounted on glass slides, deparaffinised with xylene and rehydrated to DEPC treated water through a graded series of ethanol (100%, 70%, 50% and 25%) and left in PBS for 10 min. After permeabilization in 0.1% Triton X-100 in PBS for 10 min, the sections were washed in PBS (2×5 min) and then treated with Proteinase K (10 µg/ml) during 5 min at 37°C, followed by rinsing 2×30 sec in PBS. The tissue was first post-fixed in 4% PFA (pH 9.0, 10 min), rinsed with 0.2% glycine in PBS for 30 sec, and then a second post-fixation, using EDC (1-ethyl-3-(3-dimethylaminopropyl) carbodiimide) was performed as followed: The sections were preincubated in 0.13 M 1-methylimidazole, 300 mM NaCl (pH 8.0) for 2×10 min before Bio-Rad's adhesive frames (Frame-Seal) were mounted on the slides and 500 µl of the EDC fixation solution (176 µl EDC in 10 ml 0.13 M 1-methylimidazole, 300 mM NaCl (pH 8.0)) was added to each slide and incubated at room temperature for 1.5 h. After washing in 0.2% glycine in PBS for 30 sec and PBS for 2×3 min, the sections were acetylated (5 min in 0.1 M TEA (Triethanolamine), 0.9% NaCl) followed by 5 min in the same buffer with the addition of 0.5% acetic anhydride). The slides were then rinsed in 2×SSC for at least 5 min before they were preincubated for 2 h with hybridization solution (Sigma-Aldrich, MO,USA) at 6°C under T_m_ for the probes in a humid chamber. At the same temperature, hybridization was performed using double DIG-labelled LNA™-enhanced probes (Exiqon) incubated over night with a final probe concentration of 2.5 nM for both the negative control and the test (miR-92a) probe. After stringent wash with 0.1×SSC at 4°C for 5 min, rinsed 5 min in PBS, and the endogenous peroxidase activity was quenched with H_2_O_2_ (15 min), the slides were washed 3×3 min with PBS and incubated with anti-digoxigenin AP-conjugate (Roche, Stockholm, Sweden), followed by Nitro blue tetrazolium chloride with NBT/BCIP (5-bromo-4-chloro-3-indolyl phosphate, toluidine salt) detection and finally the slides were mounted with mounting media (DAKO).

### Automated image analysis

The TMA slides were scanned using a Leica SCN400 scanner (Leica Microsystems, Milton Keynes, UK) and uploaded and archived on the web-enabled digital slide management system, SlidePath's Digital Server (Slidepath, Dublin, Ireland). The slides were evaluated on-line by a pathologist and representative tumour regions were selected for analysis. Identical areas for the slides hybridized with the negative control probe and the miR-92a probe were annotated. SlidePath's Tissue IA (Image Analysis) system was used to determine the miR-92a algorithm which includes a colour definition file to define positive staining in the cells. The output contains quantitative measurements such as staining intensity and percentage positive pixels for the selected cells.

### Statistical methods

All statistical calculations were performed using Statistical Package for the Social Sciences version 19.0 (SPSS, IL, USA). Spearman's rank-order correlation coefficient and Fisher's exact test were used to examine statistical significance of associations between miR-92a expression and other categorical variables. The Kruskal-Wallis test was used to compare medians for continuous variables. Recurrence-free survival (RFS), defined by breast cancer-specific death, distant, regional and local recurrences, was estimated using Kaplan-Meier plots and log-rank tests. A Cox proportional hazard regression model was used for multivariate analysis. The statistical significance to the migration assays were calculated using Student's T-tests. All tests were two-sided. The presented *p* values have not been adjusted for multiple testing. *P* less than 0.05 was considered statistically significant.
